# Opportunistic screening for COPD among socially marginalized patients

**DOI:** 10.1186/s12890-024-02927-9

**Published:** 2024-03-05

**Authors:** Nina Brünés, Mette Bendtz Lindstroem, Charlotte Suppli Ulrik, Ove Andersen, Marianne Lisby, Nina Skavlan Godtfredsen, Tina Leth Hansen, Charlotta Pisinger, Vibeke Graven, Kristoffer Marsaa, Laura Hohwü Thomsen

**Affiliations:** 1 Department of Quality and Patient Care, Copenhagen University Hospital – Hvidovre, Copenhagen, Denmark; 2 Department of Clinical Research, Copenhagen University Hospital – Hvidovre, Copenhagen, Denmark; 3 Department of Respiratory Medicine, Copenhagen University Hospital – Hvidovre, Copenhagen, Denmark; 4 Emergency Department, Copenhagen University Hospital – Hvidovre, Copenhagen, Denmark; 5 Department of Clinical Medicine, Faculty of Health and Medical Sciences, University of Copenhagen, Copenhagen, Denmark; 6 Research Center for Emergency Medicine, Department of Clinical Medicine, Aarhus University Health, Aarhus, Denmark; 7 Center for Clinical Research and Prevention, Copenhagen University Hospital- Frederiksberg, Frederiksberg, Denmark; 8 Department of Multidisease, Copenhagen University Hospital – North Zealand, Hilleroed, Denmark; 9 REHPA, The Danish Knowledge Centre for Rehabilitation and Palliative Care, Odense University Hospital, Nyborg, Denmark; 10 Department of Clinical Research, University of Southern Denmark, Odense, Denmark; 11 Emergency Department, Aarhus University Hospital, Aarhus, Denmark; 12 Tryg Foundation, Virum, Denmark

**Keywords:** COPD, Screening, Equality in healthcare, Nursing

## Abstract

**Background:**

Chronic obstructive pulmonary disease (COPD) is a common disease associated with premature death. Tobacco exposure is the main risk factor, but lower socioeconomic status, early life insults, and occupational exposures are also important risk factors. Socially marginalized people, facing homelessness, substance use disorder, and mental illness, are likely to have a higher risk of developing COPD, and, furthermore, experience barriers to healthcare access and consequently poorer outcomes.

**Objective:**

This study aims to assess COPD prevalence and the impact of opportunistic screening among hospitalized patients who are in contact with hospital social nurses. These patients constitute a group of patients with a high prevalence of psychiatric and somatic diseases, substance use, low life expectancy, and are socially marginalized.

**Methods:**

The present prospective longitudinal study includes a clinical examination at baseline. Participants will have spirometry done and be interviewed regarding risk factors, socioeconomic conditions, and respiratory symptoms. The 5-year follow-up assessment incorporates data from baseline and register data over the 5 years, including information on morbidity, use of COPD medication, hospital contacts, mortality, and socioeconomic factors.

**Anticipated results:**

Referral for further diagnostic work-up and management after the screening, including COPD treatment and smoking cessation support, is expected to improve survival rates. The study is still enrolling patients.

**Trial registration:**

The study is registered at ClinicalTrials.gov , NCT04754308 with study status: “enrolling”.

**Supplementary Information:**

The online version contains supplementary material available at 10.1186/s12890-024-02927-9.

## Take home message

COPD may disproportionately affect socially marginalized patients causing a substantial disease burden. By opportunistic screening, we aim to assess COPD prevalence to facilitate early diagnosis and treatment, and by that potential outcome.

## Introduction

### Background and rationale

Chronic obstructive pulmonary disease (COPD) is a progressive, life-threatening condition caused predominantly by smoking, and includes symptoms such as breathlessness and cough [1]. The gradual onset of the disease, with as many as 85% of patients being diagnosed very late in its course [2, 3], emphasizes the critical importance of early detection for reducing disease progression and, consequently, morbidity [4]. Smoking cessation is the most important intervention to prevent the disease from worsening, and access to appropriate treatment is essential for better symptom control and, by that, improved quality of life [2, 5].

In the search for better ways to identify COPD cases, scholars have explored various methods [6]. However, the studies so far have been diverse in design and outcome, hindering conclusive findings [6]. Nevertheless, a recent study from Canada which actively looked for cases, suggests that there is a potential for early diagnosis and improved long-term prognosis [7]. While routine COPD screening in asymptomatic adults is not recommended in the U.S., screening high-risk and/or symptomatic patients aligns with international recommendations [1]. In our study, we introduce case finding in expected high-risk patients admitted to Danish hospitals.

Nation-wide studies have found social inequality in the occurrence of COPD [2], and even showing a social gradient within the range of deprivation [8]. In Denmark, COPD is the disease that contributes most to social inequality in health [6]. Socially marginalized individuals in Denmark, such as those affected by homelessness, substance use, harmful alcohol use, mental illness, and poverty, have a higher disease burden and worse well-being than the general population [9]. They also have overuse of healthcare services, including emergency departments, on-call doctors, general practitioners, and higher somatic/psychiatric admission rates [10], yet despite their increased contact with the healthcare system, they have significantly higher mortality rates, as socially marginalized people die on average 17 years earlier than the background population [9].

The Danish healthcare system is 100% tax-financed, with free access to healthcare for all citizens. However, there is increasing inequality in treatment outcomes [9, 11]. With the overall aim of reducing healthcare inequality, Danish hospitals have employed social nurses.

Social nurses are registered nurses, that provide support to socially marginalized patients throughout their hospitalization, promote treatment equity, and ensure the best possible treatment outcomes [12–14]. These nurses, who have specialized knowledge and practical experience in working with socially marginalized citizens, originate from positions such as street nurses, shelter nurses, and nurses in substance maintenance treatment clinics.

Previous studies have revealed a strong association between low socioeconomic status (SES) and the presence of COPD [2]. Although individuals from socially marginalized backgrounds tend to have more frequent hospital visits and higher mortality rates [9, 10] there is a lack of available studies examining healthcare utilization, risk factors, and mortality related to COPD in patients in contact with social nurses - a patient group that represents some of the most disadvantaged individuals in Denmark. This gap in knowledge highlights the need for comprehensive investigations to facilitate a more profound understanding of the issue, as that is likely to have significant implications for global healthcare strategies aimed at addressing the multifaceted challenges faced by socially marginalized populations.

We hypothesize that socially marginalized patients in contact with social nurses, have at least a twofold higher prevalence of COPD compared to the general population, as smoking is highly prevalent in lower income groups and is adding to socioeconomic inequalities [15, 16].

### Objective

The objective of the present study is 1) to investigate and describe the prevalence of COPD among hospitalized marginalized patients being in contact with social nurses and 2) to examine the 5-year impact of opportunistic screening for COPD and referral for health care management on outcome.

## Material and methods

### Study setting

Study participants are identified during hospitalization at the participating hospitals, that is admission to emergency, medical and surgery departments, and recruited by social nurses, working at 11 hospitals within three of the five Danish regions (Capital Region of Denmark, Region Zealand, and Central Denmark Region). The hospitals include both urban and rural areas. Patient recruitment commenced on April 19th, 2021 and is still enrolling patients, with an anticipated completion of enrolment in 2024.

### Study subjects

The goal of this study is to include socially marginalized patients, who are individuals facing multiple and very challenging life circumstances such as mental health disorders, substance use disorder, and other complex issues like homelessness and involvement in criminal activities, in the target population of social nurses. This group comprises approximately 70,000 individuals in Denmark [17]. It is important to note that there is a significant utilization of healthcare services within this population, with 13% having been hospitalized in the past 3 months, and 16% having sought care from an emergency department [9]. Ultimately, it is the responsibility of the social nurse to determine whether the patient falls within the target group, resulting in a highly selected group of patients. The findings of this investigation, based on the questionnaire, will reveal whether the included patients align with the description that constitutes the target population of social nurses.

The study inclusion criteria for patients seen by a social nurse:

- Patients aged 18 years or older
- Patients able to comply with study procedures
- Patients able to understand Danish and provide informed consent

Exclusion criteria:

- Patients without a Danish civil registration number

The exclusion of patients without Danish civil registration numbers is because they only have access to acute medical care and cannot be followed up via the nationwide Danish health registries.

This study is conducted in accordance with ethical principles derived from international guidelines, including the Helsinki Declaration [9]. All participants will have to provide written informed consent.

The study protocol received approval from the Regional Ethics Committee (H-20031386, December 10, 2020) and is registered on ClinicalTrials.gov (reference NCT04754308, February 12, 2021). The collection and processing of data have been authorized by the Capital Region on behalf of the Danish Data Agency (P-2020-137).

### Study design

This is a prospective longitudinal study with 5 years follow-up from the date of inclusion.

The power calculation is based on the assumption that the prevalence of COPD will be twice the prevalence in the Danish background population (4.3%). With a power of 80% and a significance level of 0.05, a sample comprising 511 patients is required.

### Methods

The social nurses are instructed to invite consecutively all eligible patients who fulfill the following inclusion criteria: Patients aged 18 years or older, patients able to comply with study procedures, and patients able to understand Danish.

Recruitment to the study: The social nurse is informed about the admission of an individual socially marginalized patient either by hospital staff, when personally visiting the hospital departments, through telephone or patient record software, by external collaborators (e.g., substance maintenance clinics, shelters, street nurses, etc.), and/or when a patient directly contacts the social nurse.

Social nurses invite all eligible patients to participate in the study. After providing written informed consent, they interview the patients about alcohol and substance use, housing conditions, education, employment, and about respiratory symptoms, especially COPD symptoms (see Supplement for details).

**Fig. 1:**
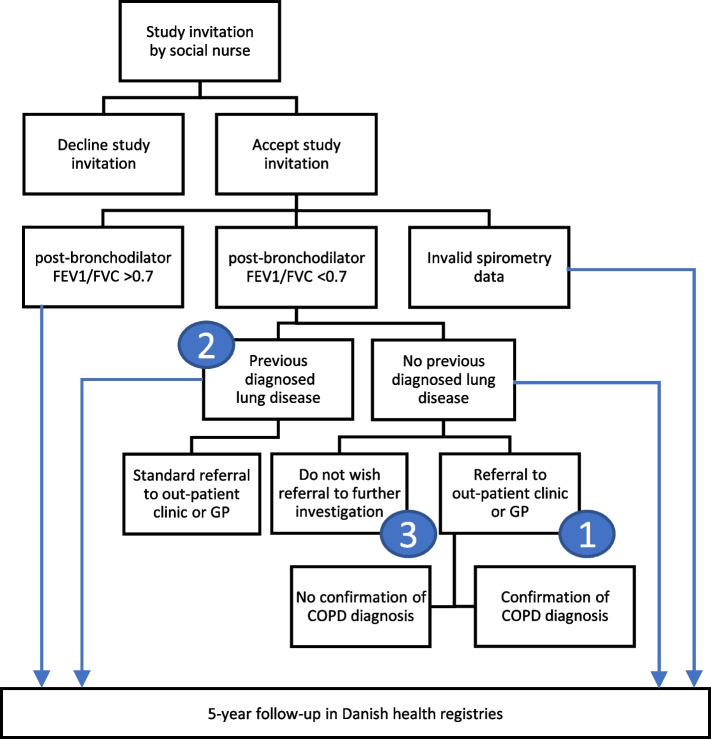
Flowchart indicating the three patient groups with COPD and the process of study-inclusion and 5-year follow-up: (1) undiagnosed COPD at inclusion who underwent further assessment, (2) previously diagnosed with COPD at inclusion, (3) COPD at inclusion who declined further assessment

Next, a spirometry is conducted by the social nurse to detect possible obstructive and/or impaired lung function (Fig. 1). It is performed using the NuvoAir device [18] corded to a smartphone/tablet with the app AIR MD. The guidelines recommend that spirometry be performed both before and after the administration of bronchodilator medications. However, due to practical reasons, the study procedure only includes post-bronchodilator spirometry, although it differs from the standard diagnostic practice. A standard dose of short-acting β2-agonist will be administered prior to spirometry.

The app AIR MD algorithm selects the best of three forced expiratory manoeuvres. The app algorithm also informs the nurse about the precision grade and if the result is acceptable.

Participants with identified impaired lung function, that is a FEV1/FVC ratio below 0.7 or the values of FEV1 and/or FVC below 80% of the predicted value, will be referred to the respiratory outpatient clinic at the hospital for further diagnostic work-up. This referral protocol is applicable irrespective of whether the participant has a pre-existing lung condition or not. In cases where it is not feasible for patients to be seen at the respiratory outpatient clinic, they are instructed to contact their general practitioner for subsequent evaluation and management.

Finally, the social nurse asks the patient about their motivation to quit smoking. In addition, they receive information about available options for smoking cessation support, including hospital resources or referrals to cost-free municipal smoking cessation services.

Coordination regarding follow-up assessment, follow-up care, and general support from social nurses constitutes standard care and is not a part of the intervention but should be taken into account in interpreting the findings.

### Outcome measures

From the baseline examination, the study will report the following outcome measures:

- Prevalence of non-reversible airflow limitation that is a post-bronchodilator FEV1/FVC ration< 0.70
- Proportion of diagnosed vs. undiagnosed COPD at the time of screening (with a previous diagnosis of COPD defined as a patient-reported diagnosis in the baseline questionnaire)
- Proportion of patients diagnosed with lung function impairment (FEV1/FVC ratio < 0.7) subsequently examined at a respiratory outpatient clinic/GP within 4 months from screening.
- Prevalence of patients diagnosed with COPD after screening (identified by ICD10 code J44, or redeemed prescriptions of COPD treatment)
- Prevalence of participating current smokers who wish to stop smoking (patient-reported outcome in the baseline questionnaire)
- Prevalence of patients with identified lung function impairment (FEV1/FVC ratio < 0.7) at screening, who have a desire to quit smoking (patient-reported outcome in the baseline questionnaire)
- Prevalence of patients who want to quit smoking (patient-reported outcome in the baseline questionnaire) and accept the smoking cessation support offers (identified by social nurse notes in patient report).

#### Five-year follow-up outcome measures

At follow-up, we will report on whether:

- Opportunistic screening for COPD by social nurses has an impact on the rate of hospital contacts. This is assessed by examining the number of hospital contacts per person year within each year of the 5-year follow-up (both outpatient visits and hospitalizations) with a diagnosis related to COPD for three patient groups: (1) the group with no previous COPD diagnosis, impaired lung function at screening, and who underwent further assessment, (2) the group of patients with previous diagnosed COPD and impaired lung function at screening, and (3) the group with no previous COPD diagnosis, impaired lung function at screening, and who declined further assessment (Fig. 1).
- Opportunistic screening for COPD by social nurses has an impact on the use of COPD medication. This is assessed by examining number of prescriptions filled for COPD medications for two patient groups: (1) the group with no previous COPD diagnosis, impaired lung function at screening, and who underwent further assessment, and (2) the group of patients with previous diagnosed COPD at (Fig. 1). The reporting of medication will be organized into distinct categories, encompassing bronchodilators, corticosteroids, rescue medications, and maintenance therapies. Within each of these groups, the dosage prescribed or dispensed will be quantified.

Previous or no previous COPD diagnosis is assessed by the questionnaire at the time of screening as a patient-reported outcome. The lung function impairment is defined as FEV1/FVC ratio < 0.7. In the reporting, patients are stratified into groups with confirmed and non-confirmed COPD diagnosis.

### Data collection

#### Baseline

Baseline data are derived from lung function measurements and the interviews and questionnaires completed at inclusion.

The data is sampled online by social nurses using a smartphone or tablet, and the data is directly entered into REDCap (Research Electronic Data Capture) [19]. Further, the results of the spirometry are uploaded directly from the smartphone to REDCap.

If online data entry is not possible, a paper version of the survey will be available. At the end of the inclusion period, they will be sent to the Department of Clinical Research at Hvidovre Hospital and entered manually into REDCap.

Upon reaching the necessary number of participants, the local investigators will refer to the outpatient bookings and physician notes in the electronic patient record, to determine whether the participants underwent further evaluation at a department of respiratory medicine and if a diagnosis of COPD was confirmed (as indicated by ICD10 coding J44).

#### Five-year follow-up

Follow-up at 5 years will be performed using the unique civil registration number (Central Person Register (CPR) number) [20] through linkage to national Danish registers. A CPR number is a unique personal identification number assigned to all residents in Denmark. The CPR number consists of a unique 10 digits and includes information about the person’s date of birth.

Data on healthcare utilisation and hospital contacts in the cohort will be obtained from the Danish National Patient Register (NPR), and mortality data will be obtained from the Cause of Death Register. Data on redeemed prescriptions for COPD medication will be obtained from The Danish National Database of Reimbursed Prescriptions.

Patients’ labour market attachment and income basis are based on extractions from the Register-based Labour Force Statistics (RAS), administered by Statistics Denmark. Data on the highest acquired level of education (HFAUDD) will be obtained from Statistics Denmark.

The CPR numbers and collected survey data from REDCap for the study participants are transferred to Statistics Denmark (DST), where they are combined with information from the Danish National Patient Register, the Cause of Death Register, and the Danish National Database of Reimbursed Prescriptions. Data processing at DST is conducted using pseudonymized data within a secure server environment.

### Variables

#### Self-reported demographic variables

Demographic variables included sex and age at inclusion. The development of COPD is strongly associated with age and will be presented as a categorical variable in the following categories: 18–24, 25–34, 35–44, 45–54, 55–64, 65–79, 80+ years.

The categories for the highest completed education are divided into the following: incomplete primary school, primary school (completed 9th or 10th grade), high school/gymnasium, vocational education, tertiary education (short/2 years, medium/3–4 years, and long/5–6 years).

The indicator regarding attachment to the labour market is divided into three categories: employed, unemployed, and out of the workforce.

Housing conditions are included to describe the degree of vulnerability and include questions related to housing situation, type of housing/rough sleeping and whether they live alone or with others.

Symptom burden and impact on quality of life in patients diagnosed with COPD is evaluated using the COPD Assessment Test (CAT) [21], a validated questionnaire specifically designed to assess various aspects of their daily respiratory symptoms and limitations in daily activities.

#### Self-reported variables related to health risk assessment

Smoking status is evaluated based on the following categories: never smoked, former smoker, occasional smoker, and current smoker. The patients’ desire to quit smoking is self-reported.

Alcohol use is assessed by the AUDIT-survey (Alcohol Use Disorder Identification Test) developed by WHO [22].

Drug use is defined as attending substance maintenance treatment or reported use of other substances.

#### Clinical variables

The patients enrolled in this study may be categorized into four groups according to their diagnostic status: those previously diagnosed with COPD (self-reported), those identified as having COPD through screening, those without COPD as determined by screening, and those with an unknown diagnostic status due to insufficient lung function test. COPD identified through screening refers to people whose post-bronchodilator FEV1/FVC ratio is less than 0,7, according to the measurements obtained by the social nurse.

When assessing the prevalence of COPD, the groups diagnosed through screening and self-reported COPD are considered. Participants who are unable to perform spirometry are classified as having an unknown diagnostic status and are not included in the calculation of COPD prevalence.

At the 5-year follow-up, the prevalence of patients diagnosed with COPD after screening is identified either through the National Patient Health registry based on the ICD10 code J.44 or by examining prescriptions for COPD medication (including all types of inhalation medicine) recorded in The Danish National Prescription Database.

The utilization of healthcare services is evaluated based on all hospital contacts, including outpatient visits, admissions, and emergency department (ED) visits.

Admission diagnosis and department will be included as descriptive variables - partly to ascertain whether there are patterns in where the screening has the greatest impact, and partly to assess whether there is selection bias in the study.

### Statistical considerations

Continuous variables will be presented as mean with standard deviation if the variable can be assumed to be normally distributed, otherwise, the median and interquartile range will be presented. Categorical variables will be presented with frequencies for each level and their corresponding percentages.

The prevalence of COPD in the study population will be compared to the prevalence in the Danish general population using an unadjusted test of the ratio of proportions, with a normal approximation of the logarithm of the ratio.

Since participants can die or otherwise drop out during the follow-up period, participants contribute with different follow-up lengths. The number of contacts in the follow-up period will therefore be reported as a rate (contact rate) of the number of contacts divided by the follow-up length, that is as contacts per year.

The healthcare contact rate will be compared among the three previously described COPD groups: undiagnosed COPD at inclusion who underwent further assessment, undiagnosed COPD without further assessment, and previously diagnosed COPD at inclusion, using a Poisson regression model. The model will include potential confounders as independent variables in addition to the COPD groups.

Sub-analyses for the rate of acute contacts and outpatient contacts will be conducted using models constructed in the same way as for all contacts.

Medication usage as a rate: number of prescriptions per year. Likewise, a Poisson regression model will be used to compare the rates among the COPD groups. However, the groups will be divided into no COPD versus diagnosed COPD. We will employ rigorous control for potential confounders. Specifically, age, smoking history, and comorbidities will be included as covariates in our models.

## Discussion

Our study is a pragmatic clinical study involving a marginalized patient population that is often excluded from participating in clinical trials due to lower health literacy and marginalization [23].

A potential methodological limitation in this study is the possibility of measurement errors arising from the use of social nurses rather than nurses with specialized respiratory training to conduct spirometry. To mitigate these risks, training is provided through local respiratory medical staff. Additionally, the social nurses are also required to be familiar with the Danish standard for lung function testing [24]. Moreover, social nurses interact directly with five to eight patients during a day shift, based on a subjective assessment of which patients need the most attention. This results in a carefully selected group of marginalized patients, potentially introducing selection bias between the study sites.

Furthermore, the handheld technical equipment used for performing the lung function test may increase the risk of measurement errors. Hence, we cannot make a definite diagnosis of COPD, but detect a possible impairment or obstructive pattern in the spirometric values. When suspicion of lung disease arises, patients undergo the standard diagnostic procedure at the current hospital, with some patients assessed in the outpatient clinic and others referred to their general practitioner. Since we cannot retrieve diagnosis from general practitioners, diagnosis verification is only available for those who attend the outpatient clinic. The five-year follow-up will determine if patients receive regular prescribed medication, facilitating confirmation of the COPD diagnosis.

We employ post-BD spirometry utilizing airflow limitation as the criterion for COPD diagnosis. Although COPD is the predominant cause of irreversible airflow limitation, it is noteworthy that a minority of patients, including those with conditions such as bronchiectasis, cannot be entirely ruled out from our study cohort. Further, an essential consideration involves the exclusion of individuals with severe, acute illnesses from the assessment of COPD prevalence due to their inability to actively participate in lung function measurements.

A strength of the study is the use of a multi-centre design on a national level, which thus with a high degree of validity reflects this subgroup of the Danish population.

The socially marginalized group is often overlooked in research [15], and this study aims to contribute to our understanding of the long-term morbidity and mortality associated with smoking in this population. By doing so, we can enhance our knowledge of the detrimental health effects of tobacco within the context of challenging social circumstances. Additionally, the collection of patient-reported outcome measure (PROM) data through the CAT form will provide valuable insights into the daily symptom burden experienced by both smokers and non-smokers within this patient group. These unique data will offer an in-depth understanding of the respiratory-related everyday experiences of socially marginalized people in Denmark. Should the study demonstrate an increased symptom burden, morbidity, and mortality associated with tobacco smoking, it might warrant further investigation into more intensive smoking cessation treatment or harm reduction strategies involving nicotine substitution.

### Supplementary Information

**Supplementary material 1.**

## Data Availability

No datasets were generated or analysed during the current study.
