# Supplementary material for: Opportunistic screening for COPD among socially marginalized patients
Source: BMC Pulm Med. 2024 Mar 5;24:113. doi: 10.1186/s12890-024-02927-9 (PMC10916054; doi:10.1186/s12890-024-02927-9)
Supplement: Supplementary file 1 — Supplementary material 1. [file 12890_2024_2927_MOESM1_ESM.docx]

Supplement
COPD among socially marginalized patients: Questionnaire and spirometry

**Lung disease**

Du you have a lung disease? Yes


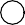

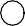

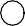


No

(diagnosed) Do not know

What lung disease?
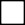
 COPD


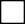
 Asthma


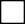
 Other (ex. Tuberculosis, Cancer)
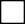
 Do not know

Do you get pulmonary medication regularly? Yes

No


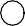

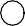


Which medication?

(Write the name of the medication or type of medication if the patient does not remember the name)

Which medication?
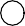
 Do not remember

**General practitioner**

Do you have your own doctor (general practitioner))? Yes No

Do not know


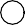

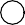

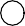


Do you use your own doctor? Yes

No


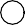

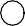


Have you had contact with your own doctor within the Yes

last year? No


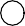

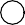

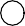


Do not know

Have you had contact with your own doctor within the Yes

past 3 months? No


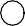

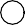

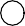


Do not know

**Smoking habits**

What are your tobacco-smoking habits? Never smoked Previously smoked

Occasional smoker (smoker, but not daily)


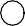

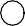

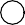

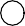


Daily smoker

How many cigarettes do you smoke daily?

What was your age when you started smoking daily?

What was your age when you quit smoking?

Do you want to quit smoking?


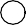
 Yes
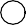
 No

Have you previously received an offer to quit smoking from Yes

doctor, hospital, municipality or others? No


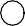

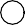


(This refers to whether the staff at hospitals, drop-in centres or the like have asked if you are interested in participating in a smoking cessation offer)

Have you previously participated in a smoking cessation program? Yes No

(formalized program)


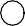

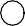


Do you want referral to a smoking cessation program?

(If the patient answers yes, measures should be taken in relation to the desired offer)
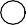
 Yes
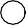
 No

Do you use e-cigarettes/vapers? Yes No


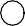

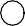


What are your cannabis smoking habits? Never

Daily


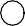

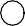

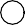

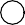

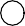


Weekly

Rarely

Former smoker

What are your smoking habits in relation to smoking heroin Never

or others drugs? Daily


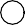

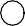

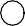

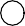

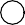


Weekly

(here the focus is on smoke from sources other than Rarely

cigarettes, vapers and cannabis) Former smoker

**Alcohol habits**

How often do you drink alcohol? 4 times a week or more
1-2 times a week

2-4 times a month Monthly or less often Never


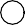

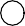

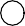

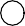

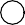


How many alcohol units do you consume on a typical 1-2


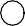

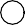

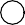

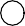

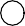


day, where you drink alcohol? 3-4

5-6

Definition of alcoholic strength: 7-9

1 alcohol unit = 1 glass of wine, 1 regular beer or a drink 10 or more

2 alcohol units = 1 strong beer

How often do you drink 5 alcohol units or more? Never Rare

Sometimes a month


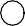

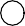

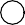

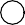


More often than a few times a month

How many units of alcohol do you drink per week (estimate)?

Helpful calculations in case of heavy alcohol consumption:

1 bottle liquor per week = 35 units per week

2 bottles liquor per week = 70 units per week

1 bottle liquor per day = 245 units per week

2 bottles liquor per day = 490 units per week 10 regular beer per day = 70 units per week 10 strong beer per day = 140 units per week

**Substances**

Are you in substitution treatment? Yes

No


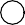

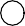


(Methadone, buprenorphin, heroin, mv.)

Do you use other substances? Never

(e.g. benzodiazepine, opioid, other) Rarely


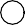

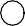

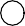

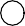

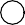

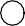


Sometimes a month

Weekly

Daily Former User

What other substances do you use?
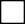
 Benzodiazepine
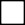
 Opioid

(Choose one or more options if necessary
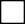
 Amphetamine
 – the amount is not important)
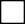
 Ritalin


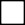
 Other

How often do you use substances other than those mentioned? Never

(e.g. nitrous oxide, lighter gas, mushrooms, LSD, etc.) Daily Weekly Rarely


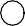

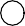

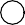

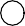


**Housing conditions**

Do you have a permanent home? Yes

(here we mean accommodation with a signed agreement, No e.g. lease agreement - i.e. not temporary residence as ex.


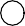

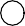


shelter)

Do you live: Alone

Alone with children


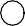

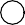

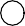

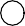

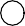

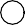

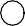

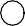


With partner

With partner and children

With parents

With parents and children

With friends/siblings or other family/others

With friends/siblings or other family/others and children

Stay overnight: On the street

In a shelter/night heating room or similar.


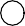

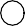

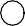


With friends/acquaintances

**Education and employment**

What highest level of education have you completed? Not completed primary school (9th grade)


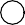

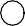

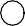

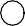

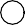

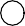

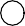


Primary school (9th or 10th grade) Gymnasium/High school

Vocational training

Short tertiary education (2 years)

Medium higher education (3-4 years)

Long higher education (5-6 years)

What is your attachment to the labour market? Working

Unemployed (incl. activation, internship, sickness benefit, rehabilitation, etc.)


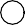

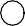


Outside the labour market (pension/early retirement) Unemployed and receiving no public benefit


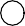

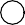


Other (write affiliation in note)

If attachment to the labour market is unclear, describe the affiliation/status here:

What job do you have?

**Height and weight**

How tall are you? (listed as cm)

(If unsure, feel free to measure if possible. Important in relation to lung function calculation)

What do you weigh? (listed as kg)

(If unsure, feel free to take steps if possible. Important in relation to lung function calculation)

**Lung function test (spirometry)**

**The following are notes in connection with the spirometry and should not be addressed to the patient**

Instruction: The spirometry is performed after taking bronchodilator medication and the following is completed

FEV1 (liters) - after bronchodilator inhalation

(Litres are given to two decimal places, e.g. 3,09)

FVC (liters) - after bronchodilator inhalation

(Litres are given to two decimal places, e.g. 3,58)

FEV1/FVC ratio after inhalation (calculation)

Test of lung function could be performed? Yes No

Was there a clinically relevant reduction of Yes

pulmonary function? No

Please refer for further investigation if FEV1 or FVC is reduced to less than 80% of expected or FEV1/FVC < 0,7

Referral to the pulmonary outpatient clinic via Yes

electronic patient record? No

Reason why no referral has been made

**CAT score - to be completed for everyone - even those who do not have impaired lung function or lung disease. For patients with no lung disease, the questions are rephrased so that the word "lung disease" is omitted.**

Are you coughing?

Select a value on the scale where 0=I never cough and 5= Icough all the time

0 1 2 3 4 5

Do you have mucus in your chest?

Choose a value on the scale where 0=no mucus at all in my chest and 5=My chest is completely filled with mucus

0 1 2 3 4 5

Does your chest feel tight?

Select a value on the scale where 0=My chest does not feel tight at all and 5 = My chest feels very tight

0 1 2 3 4 5

Are you breathless when you walk up a hill or one flight of stairs?

Select a value on the scale where 0=I am not breathless and 5 = I am very breathless

0 1 2 3 4 5

Are you limited doing any activities at home?

Select a value on the scale where 0=I am not limited and 5 =I am very limited
(In case of homelessness: are you limited in everyday activities?)

0 1 2 3 4 5

Are you confident leaving your home despite your lung condition?

Select a value on the scale where 0=I am confident and 5 =I am not at all confident

(In case of homelessness: Are you restricted from getting around because of your lung condition?)

0 1 2 3 4 5

Do you sleep soundly?

Select a value on the scale where 0=I sleep soundly and 5 =I don’t sleep soundly because of my lung condition (On homelessness: Is your sleep affected by your lung condition? )

0 1 2 3 4 5

Do you have energy?

Select a value on the scale where 0=I have lots of energy and 5 =I have no energy at all

(Explanation: Patients' subjective assessment of what they understand by energy and how they evaluate the degree of energy)

0 1 2 3 4 5

Total CAT score (calculated by REDCap):
